# Differential expression profile of gluten-specific T cells identified by single-cell RNA-seq

**DOI:** 10.1371/journal.pone.0258029

**Published:** 2021-10-07

**Authors:** Ying Yao, Łukasz Wyrozżemski, Knut E. A. Lundin, Geir Kjetil Sandve, Shuo-Wang Qiao

**Affiliations:** 1 Department of Immunology, University of Oslo, Oslo, Norway; 2 Centre for Immune Regulation, University of Oslo, Oslo, Norway; 3 K.G. Jebsen Coeliac Disease Research Centre, University of Oslo, Oslo, Norway; 4 Department of Informatics, University of Oslo, Oslo, Norway; Chittaranjan National Cancer Institute, INDIA

## Abstract

Gluten-specific CD4^+^ T cells drive the pathogenesis of celiac disease and circulating gluten-specific T cells can be identified by staining with HLA-DQ:gluten tetramers. In this first single-cell RNA-seq study of tetramer-sorted T cells from untreated celiac disease patients blood, we found that gluten-specific T cells showed distinct transcriptomic profiles consistent with activated effector memory T cells that shared features with Th1 and follicular helper T cells. Compared to non-specific cells, gluten-specific T cells showed differential expression of several genes involved in T-cell receptor signaling, translational processes, apoptosis, fatty acid transport, and redox potentials. Many of the gluten-specific T cells studied shared T-cell receptor with each other, indicating that circulating gluten-specific T cells belong to a limited number of clones. Moreover, the transcriptional profiles of cells that shared the same clonal origin were transcriptionally more similar compared with between clonally unrelated gluten-specific cells.

## Introduction

Celiac disease (CD) is an autoimmune disorder that primarily affects the small intestine. It is caused by an adverse reaction to gluten, which is a group of proteins typically found in wheat, rye and barley. The symptoms of celiac disease include diarrhea, abdominal distention, malabsorption and loss of appetite [1]. The vast majority of celiac disease patients express human leukocyte antigen (HLA)-DQ2.5 (HLA-DQA1*05/HLA-DQB1*02, expressed by 90% of patients), and the remaining patients are either HLA-DQ8 (HLA-DQA1*03/HLA-DQB1*03:02) or HLA- DQ2.2 (HLA-DQA1*02:01/HLA-DQB1*02) [2, 3]. The strong major histocompatibility complex (MHC) class II association shows that CD4^+^ T cells play an important role in the pathogenesis of celiac disease. Effector memory CD4^+^ T cells that recognize gluten peptides presented by HLA-DQ2.5 are found in the small intestinal lamina propria of HLA-DQ2.5-positive CD patients, but are absent in healthy controls [4]. In addition to CD4^+^ T cells that mostly are found in lamina propria, CD8^+^ intraepithelial T lymphocytes are vastly increased in celiac lesions. CD8^+^ T cells are thought to be responsible for mediating the tissue damage [5].

The advance of MHC multimers, typically tetramers, facilitates the identification and isolation of antigen-specific T cells by using a complex of multiple MHC molecules each covalently linked with antigenic peptides [6]. In spite of the fact that gluten-reactive T cells of CD patients respond to multiple gluten epitopes [7], a few HLA-DQ2.5-restricted gluten epitopes were reported to be commonly recognized by a substantial proportion of gluten-reactive T cells in almost all CD patients, i.e. the gluten epitopes DQ2.5-glia-α1a, DQ2.5-glia-α2 [8], DQ2.5-glia-ω1 and DQ2.5-glia-ω2 [9, 10]. In recent years, HLA-DQ-gluten tetramers carrying these four immunodominant gluten epitopes have been used to stain and visualize gluten-specific T cells directly from blood or small intestinal tissue of CD patients [11].

Single cell transcriptome sequencing, as a rapidly developing technology, has enabled the generation of transcriptomic data on the level of individual cells. The higher resolution of transcriptional data provides an unprecedented insight into cellular identity, dynamics and function, especially for rare cells.

In this study, we used HLA-DQ-gluten tetramers carrying the four immunodominant gluten epitopes to identify and sort disease-specific CD4^+^ T cells sampled from peripheral blood of four CD patients in active disease. The transcriptome of the sorted cells was then investigated by single cell RNA sequencing. We demonstrate the different transcriptional profile of gluten-specific CD4^+^ T cells from those of non-specific T cells, where gluten-specific T cells appear as recently activated effector memory T cells with features of both Th1 and follicular helper T cells. We also show that the transcriptomes of clonally related cells are more similar than comparison of transcriptomes of clonally unrelated T cells.

## Methods

### Cell staining and sorting

Patients with untreated celiac diseases who visited Oslo University Hospital for diagnostic work-up were enrolled to the study (S1 Table). The study was approved by Regional Committee for Medical and Health Research Ethics South-East Norway (2010/2720). All patients gave written informed consent.

From each patient we collected 100 ml peripheral blood. Peripheral blood mononuclear cells (PBMC) were isolated by density gradient separation with Lymphoprep (Stemcell technologies). Tetramer staining was performed largely according to a previously published study [11]. In short, PBMC were stained with a mix of four PE-conjugated HLA-DQ2.5:gluten tetramers representing the four immunodominant gluten T-cell epitopes; DQ2.5-glia-α1a, DQ2.5-glia-α2, DQ2.5-glia-ω1 and DQ2.5-glia-ω2, and then subjected to enrichment of tetramer-stained cells. The samples were also stained with antibodies for surface markers: CD62L-PerCP/Cy5.5, CD14-Pacific Blue, CD19-Pacific Blue, CD56-Pacific Blue and integrin-β7-APC (Biolegend); CD38-FITC, CD11c-Horizon V450, CD4-APC-H7 (BD Biosciences); CD45RA-PE-Cy7 and CD3-eVolve605, (eBioscience). Pacific Blue/Horizon V450-labelled antibodies together with LIVE/DEAD marker fixable violet stain (Invitrogen) were used to exclude unspecific binding (dump channel). Live, single, CD4^+^ effector memory T cells that were CD3^+^, CD11c^-^, CD14^-^, CD19^-^, CD56^-^, CD8^-^, CD4^+^, CD45RA^-^ and CD62L^-^ were sorted as either tetramer-positive or tetramer-negative cells (S1 Fig) in alternating columns of 96-well plates (Bio-Rad) prefilled with 4 μL TCL lysis buffer (Qiagen) supplemented with 1% β-Mercaptoethanol (Sigma). Sorting was performed on an Aria II cell sorter (BD Biosciences) at the Flow Cytometry Core Facility at Oslo University Hospital. Flow cytometry data was analyzed with FlowJo software (FlowJo LLC).

### scRNA-seq library with SMART-seq2 protocol

Smart-Seq2 protocol was performed on single sorted cells as described [12]. In short, RNA was extracted from sorted single cell lysates using 2.5X of Agencourt RNAClean XP beads (Beckman Coulter). After binding and washing, beads were resuspended with 4.02 μL of Oligo Mix: 0.8 μL dNTP (10 mM, Thermo Scientific), 0.08 μL of 100 μM RT primer (Bio-TTAAGCAGTGGTATCAACGCAGAGTCGACTTTTTTTTTTTTTTTTTTTTTTTTTTTTTTVN) and 2.84 μL H_2_O. After denaturation at 72°C for 3 minutes, samples were cooled and 3.99 μL of the following RT Mix was added: 1.6 μL 5X First Strand Buffer (Invitrogen), 1.6 μL Betaine (5 M, Sigma), 0.05 μL MgCl_2_ (1 M, Sigma), 0.2 μL DTT (100 mM, Invitrogen), 0.3 μL RNase Inhibitor (40 U/μL, New England BioLabs), 0.08 μL of 100 μM template-switch oligo (Bio-AAGCAGTGGTATCAACGCAGAGTACrGrG+G, Exiqon) and 0.16 μL SMARTscribe Reverse Transcriptase (100 U/μL, Clontech). The reverse transcription condition was 42°C x 90 min, followed by 10 cycles of (50°C x 2 min and 42°C x 2 min) and inactivation at 70°C x 15 min. After cooling, the following cDNA Amplification Mix was added: 10 μL 2X KAPA HiFi Ready Mix (KAPA Biosystems), 0.02 μL of 100 μM PCRfwd primer (AAGCAGTGGTATCAACGCAGAGT) and 1.98 μL H_2_O. The reaction was carried out with 98°C x 3 min, followed by 23 cycles of (98°C x 20 sec, 67°C x 15 sec and 72°C x 6 min) and a final extension at 72°C x 5 min. PCR products were purified by using 1X Agencourt RNAClean XP beads, and amplified cDNA were eluted in 10 μL H_2_O. Selected samples were assessed on 2100 Bioanalyzer using High Sensitivity DNA Kit (Agilent Technologies). Expected single-cell cDNA concentration varied between 0.2–1 ng/μL with size distribution peaking around 2 kb.

Tagmentation was performed as described [13]. The plasmid encoding Tn5 transposase was a generous gift from Rickard Sandberg and Gosta Winberg, Karolinska Institute. Tn5 protein was expressed and purified in our laboratory according to [13]. The tagmentation reaction consisted of mixing 3 μL of 0.4–1 ng cDNA with 4 μL of freshly prepared TAPS-DMF buffer (Sigma and Fluka respectively) and 1 μL of Tn5-oligo transposome complex (0.4 μM). The 8 μL reaction was incubated at 55°C x 5 min, cooled and then quenched with 2 μL of 0.2% SDS (Sigma-Aldrich) for 5 minutes. The libraries were amplified with the following Post-Tagmentation PCR Mix: 4 μL 5X KAPA HiFi Buffer, 0.6 μL dNTP Mix (10 mM), and 0.4 μL KAPA HiFi Polymerase (1 U/μL) (all from Kapa Biosystems); and 2.5 μL of 1 μM column A-H/a-h primer and 2.5 μL of 1 μM row 1-12/13-24 primer. The PCR reaction was carried out with 72°C x 3 min and 95°C x 3 min followed by 12 cycles of (95°C x 20 sec, 55°C x 30 sec and 72°C x 30 sec) and final extension at 72°C x 5 min. PCR products were purified with 13 μL (0.65X) of AMPureXP SPRI beads (Beckman Coulter) and eluted with 10 μL H_2_O. Selected tagmentated/barcoded samples were assessed for size distribution (between 175–1000 bp and peaking around 300 bp) and concentration (0.5–2 ng/μL) on Bioanalyzer. Single-cell samples from the 96 wells were pooled, extracted with 1.8X of AMPureXP SPRI beads, and eluted with 40 μL of EB buffer. Sample concentrations were around 20 ng/μL.

Samples were sequenced at the Norwegian Sequencing Centre (Oslo University Hospital), on the NextSeq500 platform with a 150 bp kit, high output mode, with paired end reads. 90 bp were sequenced in Read 1 and 60 bp in Read 2. In total, amplified cDNA from 1023 sorted single cells from four untreated CD patients were sequenced.

### Quantification of genes

Paired-end reads were mapped to the human reference genome GRCh38 containing alternative loci with gene annotations curated by Ensembl release 86 using Salmon [14] version 0.7.2 with parameters -l UIuse-VBOpt-numBootStraps 30–seqBias–gcBias. The quasi-mapping index in Salmon was built using a default k-mer length of 31. Read counts of transcripts were then aggregated to gene level. All sequencing data were processed on the TSD (services for sensitive data) platform owned by the University of Oslo, operated and developed by the TSD-service group. Further analysis of extracted gene expression matrices was performed using R version 3.4.4.

### Cell and gene selection

Based on the overall distribution of all single cell libraries (S2 Fig), the following criteria were applied for quality control: mapping rate >30%; number of reads > 100 000; number of detected genes 1 800–15 000; proportion of reads mapped to mitochondrial genes < 12.5%. From total 1 023 T cells analyzed, 739 passed all four quality control criteria and were included in the subsequent analyses (S2 Table). By excluding genes expressed in less than 10 cells that have passed the quality control, 24 121 genes from 739 cells were used in the study.

### Data integration and visualization of the cells from four CD patients

After full-quantile normalization [15], log-transform and scaling on the gene count matrix, we followed a pipeline in Seurat 3.0.2 [16] to integrate the datasets from four different batches, one patient each batch. In this process, the FindIntegrationAnchors function was used to identify pairs of mutual nearest neighbor cells, serving as “anchors” to guide integration. Based on the expression of top 10 000 highly variable genes in each dataset, 224 to 613 anchors were identified between any two of the four datasets by using top 30 dimensions in CCA (canonical correlation analysis) and 100 neighbors when filtering the anchors. Based on the number of anchors, the similarity between the pairwise datasets were calculated. The datasets were then iteratively integrated in the order of the similarity levels using IntegrateData function with default parameters. Principal component analysis (PCA) was performed on the integrated data. The top five principle components (PCs) were used as input features for running uniform manifold approximation and projection (UMAP). Finally, the integrated data was visualized in the two-dimensional UMAP plot. We produced UMAP plots split by patient and plate (S3 Fig) to ensure that batch effect was corrected by data integration.

### Data integration of public scRNAseq data of CD4+ T cells from healthy individuals

We included the single cell transcriptomic data of PBMC cells from two human healthy donors, GSM4138162 and GSM4138163 from GSE139324 of NCBI [17]. Cells were preselected according to the following criteria: number of reads > 2400, number of detected genes between 850 and 2000, proportion of reads mapped to mitochondrial genes < 12.5%. R package SingleR [18] and reference databases: Human Primary Cell Atlas [19], Blueprint [20] and ENCODE [21] were used for cell type annotation. Based on the result, 225 cells including 141 cells from the 1^st^ healthy donor (GSM4138162) and 84 cells from the 2^nd^ healthy donor (GSM4138163), were identified to be effector memory cells with high scores of confidence. The data was integrated with the 739 cells from the four CD patients in our study using the same method as described, where top 2000 highly variable genes repeatedly variable across datasets were selected to find sets of anchors. The top eight principle components (PCs) were used as input features for running uniform manifold approximation and projection (UMAP).

### Differential expressed genes and gene set enrichment analysis

The differential expression analyses comparing tetramer-positive and tetramer-negative cells were performed using MAST embedded in the Seurat package. P-values were adjusted for multiple testing using false discovery rate (FDR). Fold change (FC) of gene expression was converted to log2 base (log2FC). Gene set enrichment analysis was performed by GSEA 4.0.1 using the entire result of the above test for differentially expressed genes, which included 24 121 genes ordered by the product of the sign of log fold change and log10-transformed P value calculated from the test. In this way, the most significantly upregulated gene in tetramer-binding T cells was at the top, while the most significantly down-regulated gene was at the bottom. From all the 16 746 pathways in human genome ontology biological process (GOBP) [22] and Reactome database [23], we selected for testing 4,818 pathways where the gene set size were between 15 and 500.

### Reconstructing the full length T-cell receptor

We used TraCer 0.4.0 [24] to reconstruct the full-length T cell receptors (TCR) from the transcriptome data, where multiple modules were selected in its pipeline including bowtie2 version 2.2.9, Trinity 2.0.6, NCBI-IgBlast 1.5.0 and Kallisto 0.43.0. All the other settings were the same as default.

### Comparing transcriptome similarity between clonally related and not related cells

From cells that had passed quality control and had at least one TCR chain successfully reconstructed by TraCer, we enumerate all possible pairs of cells with the same reconstructed TCR as an intra-clone dataset. Likewise, a random-clone dataset of the same size was generated by randomly selecting tetramer-positive T cells from the same patient. For each pair of cells in the two datasets aforementioned, the Pearson correlation of gene expression were calculated where the expression of TR genes was excluded. Welch Two Sample t-test was applied to test if the correlations for the intra-clone pairs were equal to those for random-clone.

## Results

### Tetramer-binding gluten-specific T cells show distinct gene expression profile

To investigate the heterogeneity of the CD4^+^ T cells at the transcriptional level, we performed single cell RNA-seq using the SMART-seq2 protocol on 1 023 cells sampled from peripheral blood of four CD patients in active disease. Effector memory (CD62L^-^CD45RA^-^) CD4^+^ T cells were sorted as either gluten-specific T cells that bound to HLA-DQ2.5:gluten tetramers presenting one of the four immunodominant gluten epitopes, hereafter denoted as tetramer-positive cells, or effector memory T cells that were tetramer-negative (S1 Fig). The majority (58–94%) of tetramer-positive cells were positive for both β7-integrin and CD38 indicating that these were recently activated T cells of intestinal origin. Since the four immunodominant epitopes used in the tetramers only capture less than half of all gluten-specific T cells, some gluten-specific T cells may also be expected to be found in a very small proportion of the control cells, primarily within the 24–34% β7^+^CD38^+^ tetramer-negative cells (S1 Fig).

**Fig 1:**
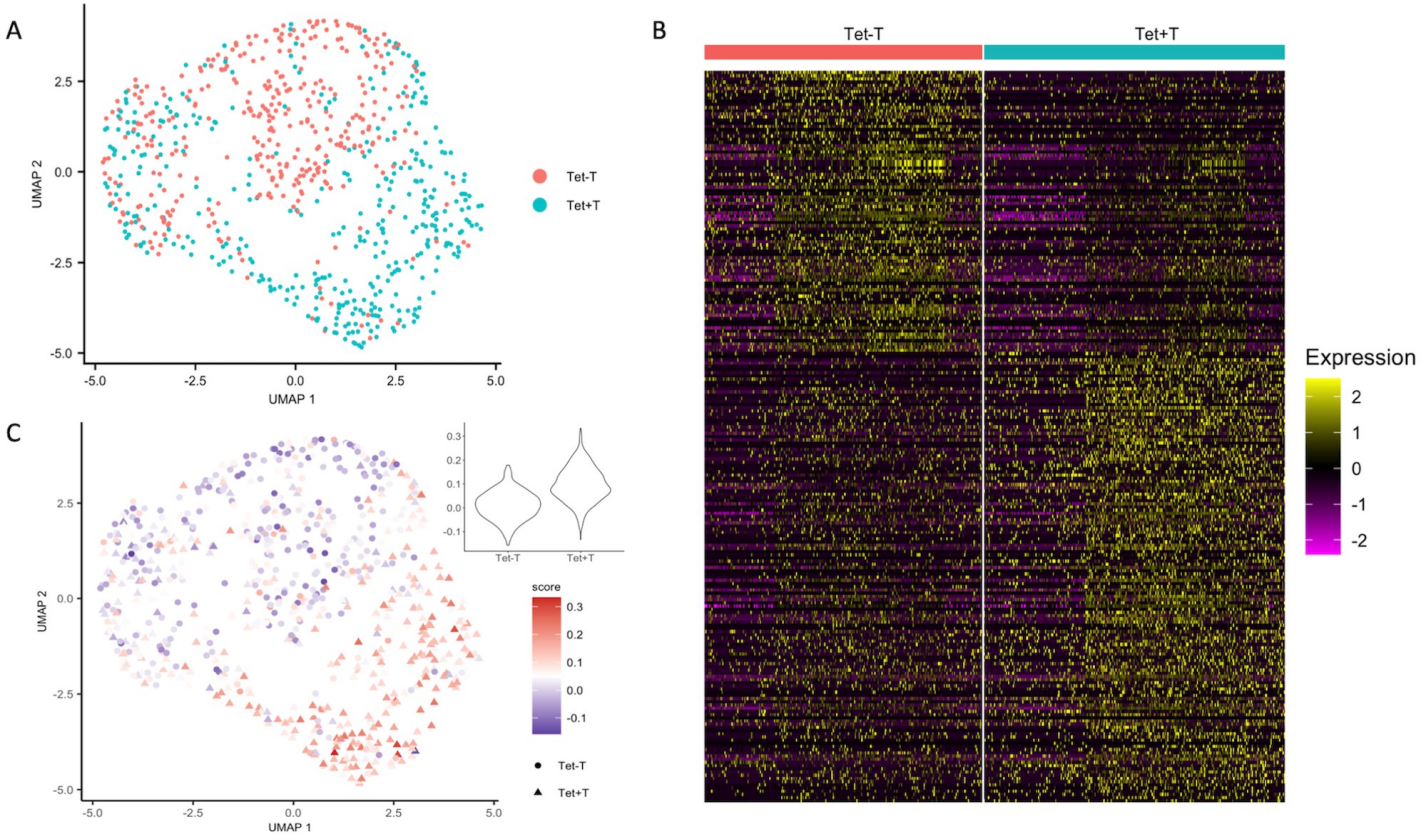
Tetramer-positive T cells show expression profiles distinct from tetramer-negative T cells. (A) UMAP plot of 739 cells from peripheral blood of four untreated celiac disease patients, colored by cell specificity. Tet+: sorted HLA-DQ2:gluten-tetramer-positive effector memory CD4^+^ T cells; Tet-: tetramer-negative effector memory CD4^+^ T cells (B) Heatmap of the 739 cells grouped by cell specificity. Features include 237 genes identified by differential expression analysis with FDR < 0.05 and absolute average fold change > 1.5. The color represents the scaled value of gene expression, where each gene has a mean close to 0 and a standard deviation of 1. (C) UMAP plot of 739 cells from peripheral blood of four untreated celiac disease patients, colored by signature scores based on differentially expressed genes reported from bulk analysis [25].

After quality control and data integration, we visualized the cells in UMAP plot (Fig 1A). The cell population clustering was to a large degree in accordance with the cell specificity. Moreover, the discordant cells were mostly located close to the junction of the two clusters.

**Fig 2:**
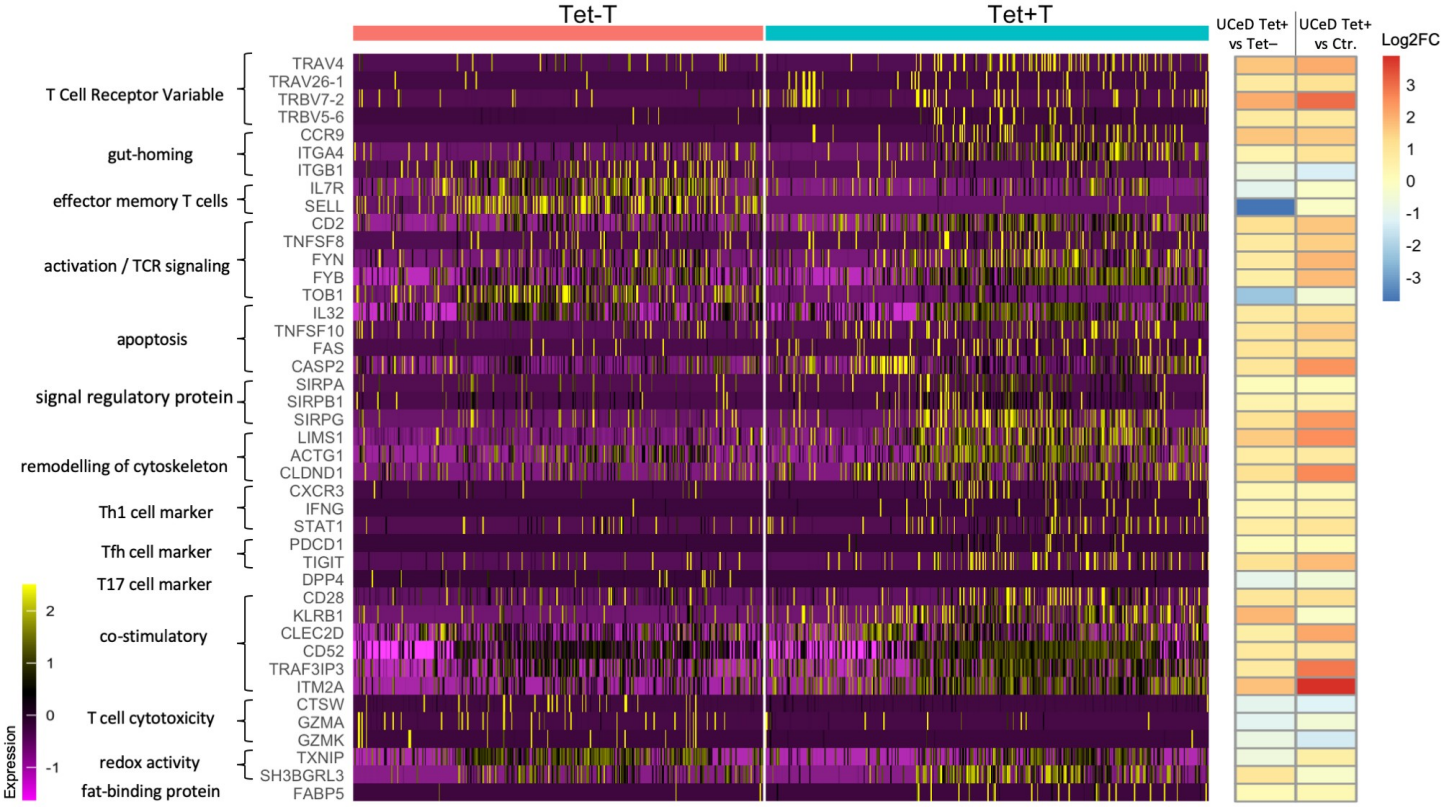
Heatmap of selected genes organized by function. The color represents the scaled value of gene expression, where each gene has a mean close to 0 and a standard deviation of 1. All genes listed are differentially expressed with FDR < 0.05. On the right, the 1^st^ column of log2FC represents the average log2 fold change expression of the genes for tetramer-positive versus tetramer-negative effector memory CD4^+^ T cells from CD patients. The 2^nd^ column represents the average log2 fold change expression of the genes for tetramer-positive effector memory CD4^+^ T cells from CD patients versus effector memory CD4^+^ T cells from healthy donors.

We identified 237 significantly differentially expressed genes (DEGs) between tetramer-positive and tetramer-negative T cells, with a false discovery rate (FDR) of below 0.05 and absolute average fold change larger than 1.5 (Fig 1B and S3 Table). Next, we wanted to see whether the results of our single-cell RNA-seq data from PBMC were consistent with the results from a bulk RNA-seq study where gluten-specific T cells were sorted with the same tetramers from duodenal biopsies of five celiac disease patients [25]. In the bulk RNA-seq study, 318 genes were identified as differentially expressed (DEGs) between the tetramer-positive and tetramer-negative cells by using DESeq2 with an adjusted P value smaller than 0.01. We used the VISION pipeline [26] and created a gene signature using the DEGs in the bulk study, where the up-regulated genes were given a positive score, and down-regulated ones were given a negative score. By projecting the single cell expression matrix to the signature, a score for each cell was calculated by summing over scores for all bulk DEGs and weighted by the expression in the single cell study. Using this approach, we found a good consistency between our scRNA-seq and the bulk RNA-seq data. The tetramer-positive cells in our data scored higher, showing that the expression pattern of the DEGs found in the bulk study were different between the tetramer-positive cells and the tetramer-negative cells in our study (Fig 1C). In addition, we integrated public scRNAseq data of circulating effector memory CD4+ T cells of two healthy individuals in study [27]. Unsupervised UMAP clustering showed that cells from the healthy controls were largely found in close vicinity with tetramer-negative T cells from untreated CD patients in our data (S4 Fig). More importantly, the changes in gene expression between tetramer-positive and–negative cells in our data are consistent with gene expression changes between tetramer-positive cells from CD patients and cells from healthy subjects (Fig 2).

Among the most up-regulated genes in tetramer-positive T cells in our single cell study, there were several T-cell receptor V-genes (Fig 2 and S5 Fig). These V-genes, such as *TRAV4*, *TRAV26-1*, *TRBV7-2* and *TRBV5-6*, have all been previously found to be preferentially used by gluten-specific T cells in CD patients [28]. This confirms the specificity of tetramer-aided sorting strategy, as well as the ability of discovering genes associated with gluten-specific T cells in our study.

### Enrichment of genes associated with T-cell signaling

**Fig 3:**
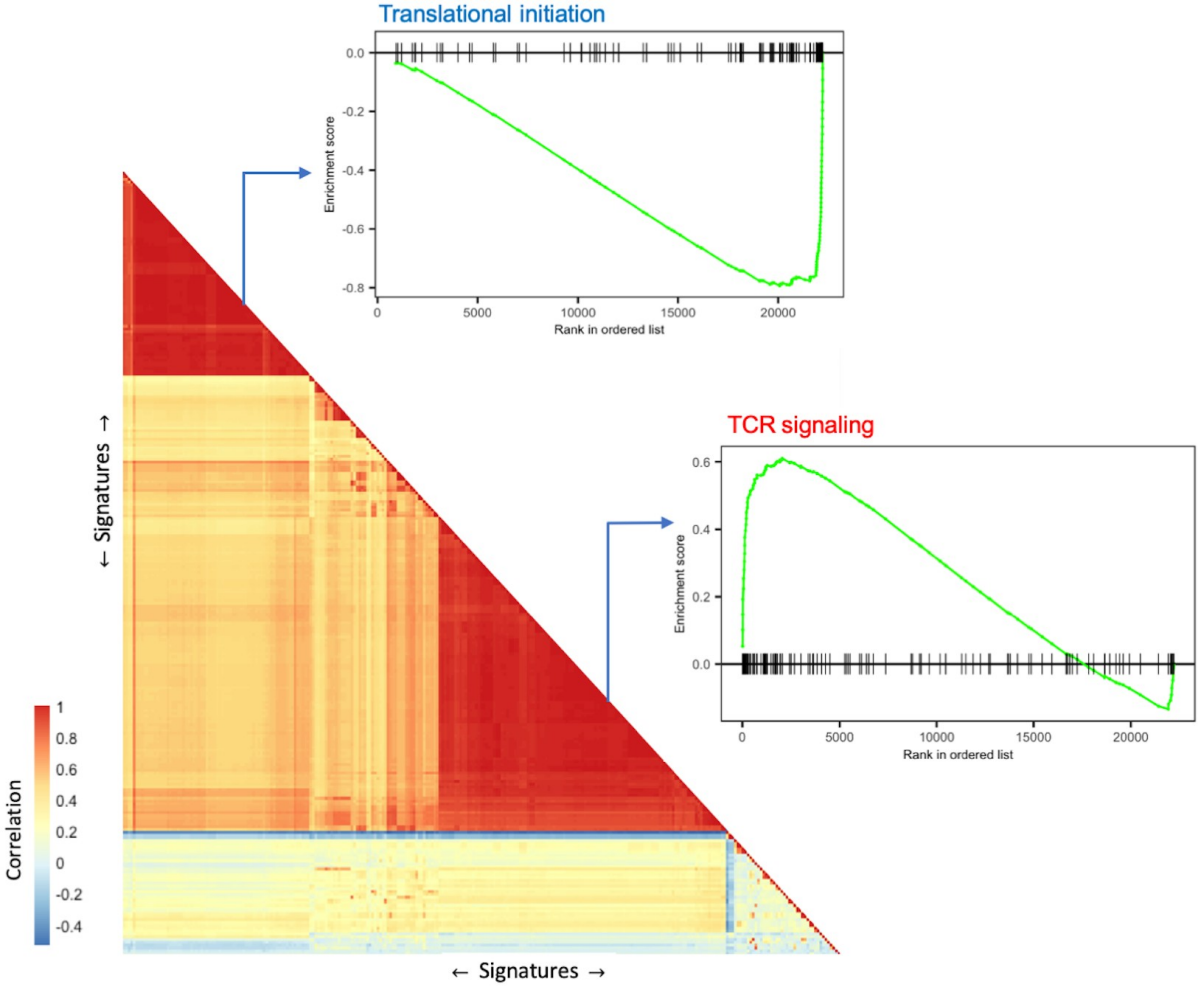
Significantly enriched signatures (gene sets) clustered based on the correlation of signature scores produced by VISION. For each of the two main clusters (one down-regulated and one up-regulated), an enrichment plot was provided for one selected signature with both high significance in gene set enrichment analysis and high level in the hierarchical database structure, e.g. the Reactome or GO biological process hierarchy.

To investigate the functional network of genes that are differentially expressed in our scRNA-seq data, we performed gene set enrichment analysis using GSEA 4.0.1 [29]. The significantly enriched gene sets with an FDR lower than 0.05 was used as signatures in VISION [26], where a signature score was calculated for each cell and each gene set. The gene sets were clustered based on the correlation of their per-cell signature scores to illustrate patterns of variation (Fig 3). The top enriched gene sets in the largest cluster were mostly associated with response mediated processes, such as TCR signaling, downstream TCR signaling; together with a small cluster of gene sets associated with co-stimulatory signal during T cell activation, such as CTLA4, CD28 family, all of which verified an increased T-cell activation in tetramer-binding T cells. Moreover, an increased signal of cytokines was observed in the tetramer-positive T cells, including the production of interleukin (IL)-10, IL-1, IL-12, IL-8, and especially cellular response to interferon-gamma (Fig 3 and S4 Table).

On the other hand, over a third of most down-regulated genes (32 of 93 genes with at least 50% decrease) in tetramer-positive cells were genes encoding ribosomal proteins. Accordingly, a cluster of gene sets associated with translation catabolic process were the top down-regulated gene pathways, indicating reduced translation and protein synthesis activities in tetramer-positive T cells (Fig 3 and S4 Table).

### Gluten-specific T cells are activated effector memory cells with helper functions

In tetramer-positive cells, we found marked up-regulation of genes associated with gut-homing, such as *CCR9* and α4 integrin (*ITGA4*), combined with the down-regulation of β1 integrin (*ITGB1*) (Fig 2). This is in accordance with the flow staining data where >90% of tetramer-positive cells were β7 integrin-positive, indicating that almost all tetramer-binding cells express the gut-homing marker of α4β7.

As a group, tetramer-positive cells showed gene expression pattern associated with effector memory T cells (S6 Fig) by using gene signature from [30], such as down-regulation of both CD127 and CD62L, encoded by the *IL7R* and *SELL* gene, respectively (Fig 2). The tetramer-positive cells showed also features of activation, such as the marked up-regulation of *CD2*, HLA class II molecules, CD30L (*TNFSF8*), and *FYN* and *FYB* kinase molecules that are involved in TCR signaling, as well as the strong down-regulation of *TOB1* (Fig 2), a pre-requisite for T-cell activation [31]. We observed marked upregulation of the pro-inflammatory cytokine IL-32, as well as upregulation of the apoptosis-inducing TNF superfamily cytokine TRAIL (encoded by *TNFSF10*), the apoptosis receptor FAS, and caspase 2 (*CASP2*). Interestingly, we also found marked up-regulation of several members of the signal regulatory protein family, including *SIRPA*, *SIRPB1* and *SIRPG*. In CD8^+^ T cells, SIRPα expression defines a subset of T cells that despite exhaustion, still remain functional [32], whereas engagement of SIRPγ on CD4^+^ T cells results in enhanced antigen-specific T-cell proliferation [33]. We found up-regulation of genes (*LIMS1*, *ACTG1*, *CLDND1*) that indicate remodeling of the cytoskeleton, possibly as an effect of activation of the tetramer-positive cells (S3 Table).

**Fig 4:**
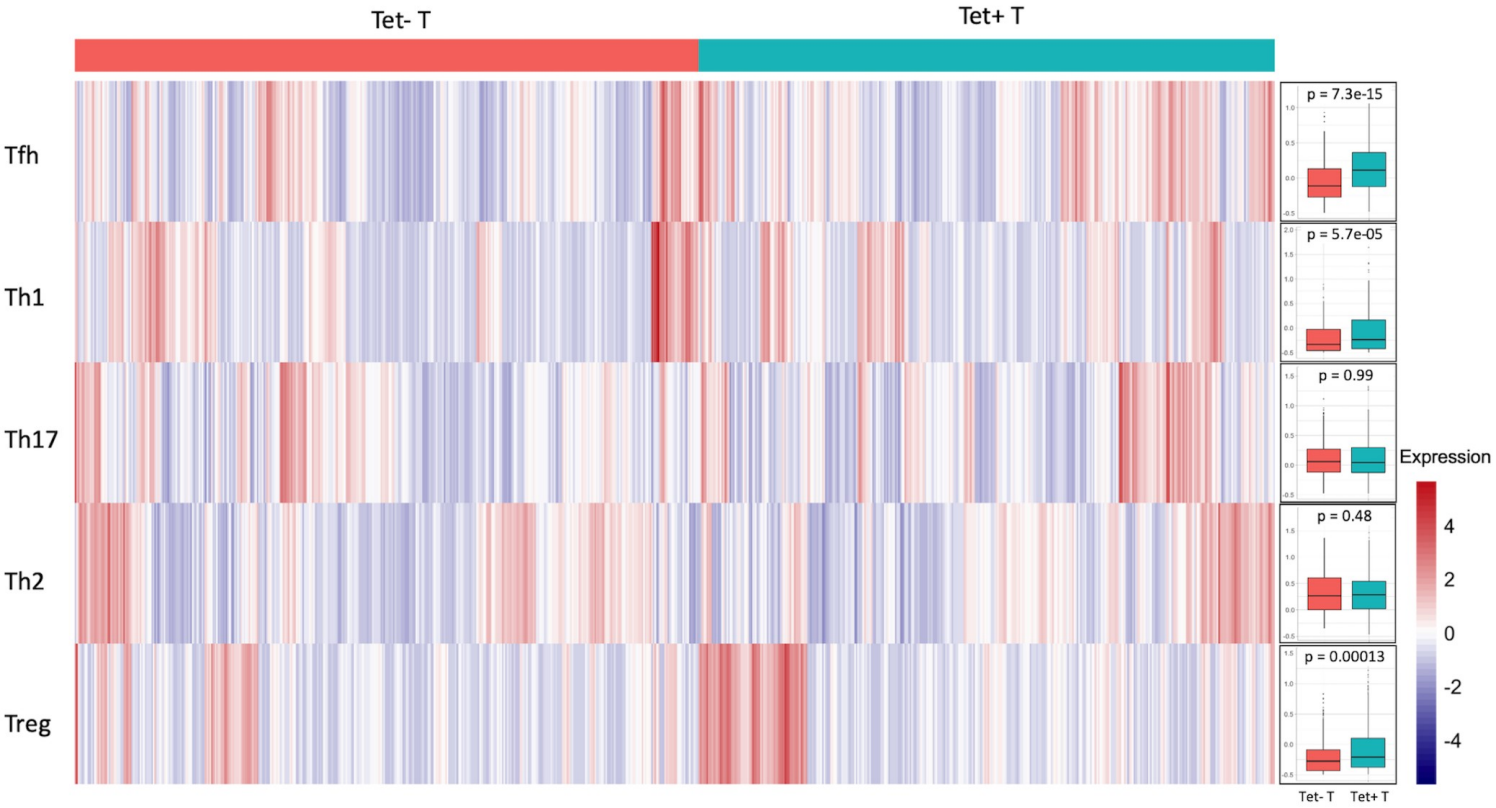
Heatmap indicating subtype of T helper cells. The heatmap of 739 cells was colored by VISION score for signature indicating follicular helper T cell (Tfh), T helper cell 1 (Th1), T helper cell 17 (Th17), T helper cell 2 (Th2), and regulatory T cell (Treg), where cells with higher score colored in red have higher expression of a certain gene signature, e.g. Tfh cell. The gene sets used for calculating the scores were the same as used in [34, 35] (S5 Table). Signature scores for cells were summarized by cell specificity in the box plot. P values were calculated by using the Wilcoxon test.

To explore the feature of tetramer-positive T cells, we applied VISION [26] to calculate scores for each gene signature, including Th1, Th2, Th17, Tfh and Treg, as used in [34, 35] (S5 Table). In accordance with the recently published mass cytometry results of gut-residing tetramer-positive T cells [25], we found that the tetramer-positive cells harbor an expression profile that primarily resembles those of Th1 cells and follicular helper T cells, as well as regulatory T cells (Fig 4). *CXCR3*, *IFNG*, *STAT1* and *PDCD1* (encoding PD-1), as well as *TIGIT*, were up-regulated in tetramer-positive cells (Fig 2). In comparison, the tetramer-positive cells did not display Th2 or Th17 phenotype, although a small subset of tetramer-positive cells displayed Treg profile, consistent with earlier reports [36].

In addition, we found marked up-regulation of genes that may have co-stimulatory effects, such as CD28, CD161 (*KLRB1*) and its ligand LLT-1 (*CLEC2D*), *CD52*, *TRAF3IP3*, as well as *ITM2A* (Fig 2) that is a gene known to be up-regulated during thymocyte selection and T cell activation [37]. On the other hand, genes associated with T cell cytotoxicity, such as cathepsin W (*CTSW*) and granzymes A and K (*GZMA*, *GZMK*) were markedly downregulated (Fig 2). Interestingly, we found that two genes that regulate the redox activity, *TXNIP* and *SH3BGRL3*, were differentially regulated in tetramer-positive cells, likely as a downstream effect of TCR signaling. This is accompanied by metabolic changes such as the up-regulation of fat-binding protein 5 (FABP5). FABP5 has been previously shown to play a critical role in the maintenance, longevity and function of CD8^+^ tissue-resident memory cells, where these cells use exogenous free fatty acids to mediate protective immunity in tissue [38].

### Clonally related cells are transcriptionally more similar

**Fig 5:**
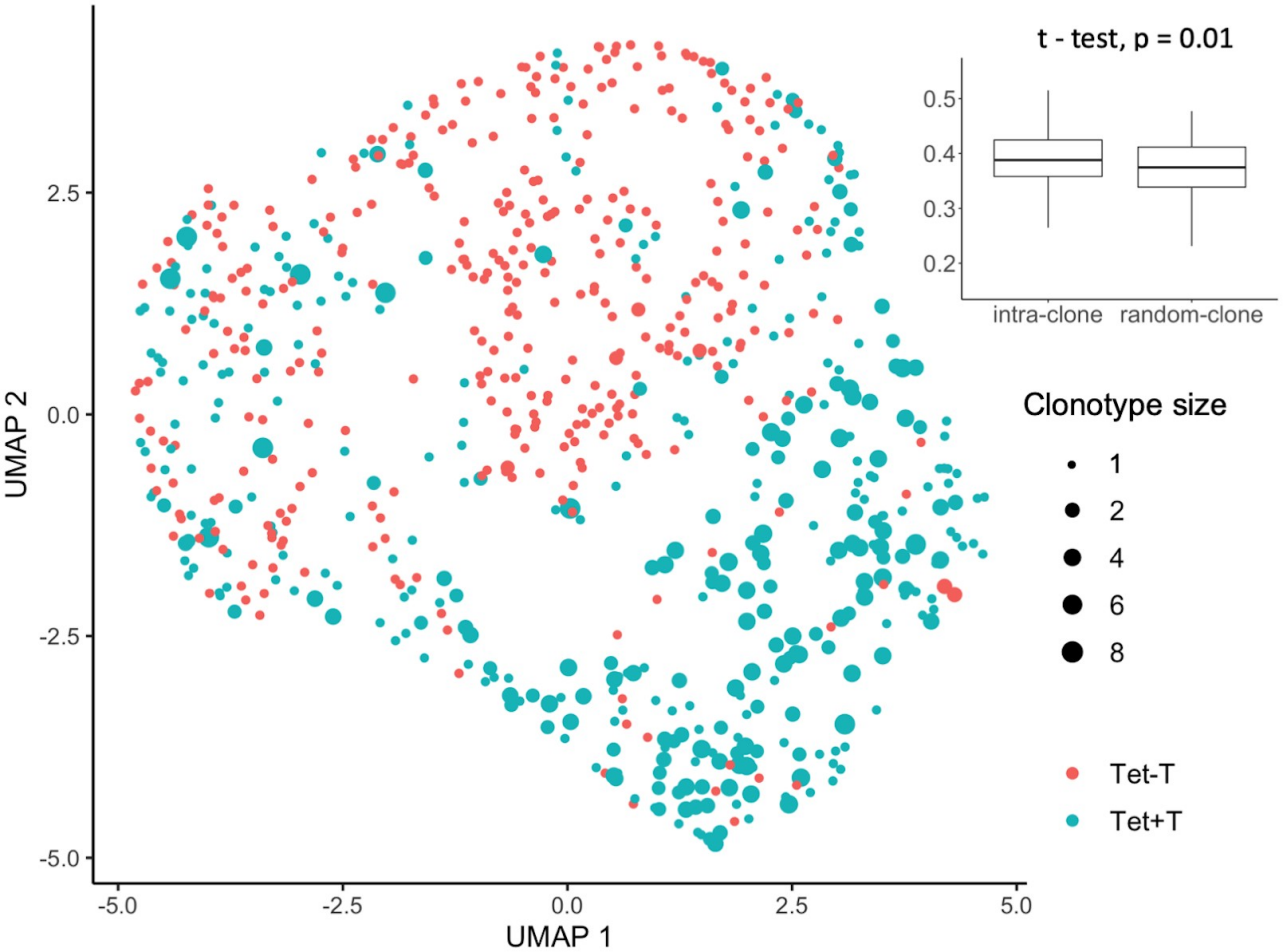
Many tetramer-positive cells were clonally expanded and clonally-related cells were transcriptionally more similar. Cells in the UMAP plot were labeled with the size of the clone to which the cell was assigned to. In the inserted box plot, values of Pearson correlation were summarized for all intra-clone and random-clone pairs.

Since our single cell RNA-seq data by SMART-seq2 contained reads spanning the entire length of the transcripts, we could use TraCer [24] to reconstruct full length TCR from the transcriptome data. Out of the 739 single cell libraries from four patients, at least one productive TCRα or TCRβ chains were reconstructed in 615 cells (83%) (S6 Table). Out of the 739 cells that passed quality control, 51 expanded clonotypes representing 160 cells were detected, as well as 455 singletons and 124 cells with no successfully assembled TCRs (S2 Table). As expected, the vast majority of cells with detected clonal expansion were tetramer-positive T cells except for three small clones of two cells each that were found among tetramer-negative cells (Fig 5 and S2 Table).

Given the clonal expansion observed in the tetramer-positive T cells, we tested if cells belonging to the same clone were transcriptionally similar. The Pearson correlation of gene expression of two cells from the same clone (intra-clone) were found to be significantly higher (t = 2.57, df = 444.9, p-value = 0.01), compared to correlation of two cells from randomly selected clonotypes (random-clone) (Fig 5), showing that cells that share the same clonal origin display more similar transcriptomic profiles.

## Discussion

Gluten-specific CD4^+^ T cells are the key drivers for the pathogenesis of celiac disease. These cells are activated when the patients consume gluten, and gluten-specific CD4^+^ T cells provide helper functions to both gluten-specific B cells and B cells that are specific to the autoantigen tissue transglutaminase (TG2). We conducted unbiased full transcriptomic profiling of circulating gluten-specific CD4^+^ T cells from four untreated celiac disease patients by single-cell RNA-seq. Compared with non-specific cells, these cells showed a more activated phenotype and carried gut-homing markers indicating that these were T cells that had been recently activated in the gut.

Gluten-specific T cells were sorted as HLA-DQ2.5:gluten tetramer-positive effector memory CD4^+^ T cells. Effector memory CD4^+^ T cells in the same sample that were tetramer-negative were used as control cells. It is possible that the binding of TCR by the tetramers could lead to some signaling events that may skew the transcriptomics data *in vitro*. However, we believe that this is unlikely to explain the strikingly different transcriptome profiles we observed. In the absence of other co-stimulatory signals, TCR binding on its own by the tetramers would lead to only weak activation, if any. We observed a wide range of activation signals that indicated *in vivo* activation by both TCR and co-stimulatory signals. The fact that transcription of the early activation marker CD69 was markedly down-regulated in our tetramer-positive cells supports *in vivo* activation. CD69 is normally up-regulated as early as half hour after adequate T-cell stimulation and then is rapidly down-regulated [39]. To note, in addition to being a classical early activation marker, CD69 is also a marker of tissue retention. T cells residing in the intestinal lamina propria are uniformly positive for CD69 [40] where CD69 suppresses the tissue egress function of Sphingosine-1-phosphate receptor 1 (S1P1) [41]. The fact that CD69 is strongly down-regulated in our tetramer-positive cells indicates that these cells may have recently egressed from the intestinal tissue, for which the down-regulation of CD69 is a prerequisite.

Early studies have shown that gluten-specific cells predominantly secrete IFN-γ upon *in vitro* stimulation [42], although more recent studies using multiplex cytokine assays showed that in addition to IFN-γ, gluten-specific T cells secrete *in vitro* a multitude of different cytokines including IL-2, TNF-α, IL-10 and IL-4 [43]. Notably, when analyzed on the single-cell level by tetramer staining and intracellular cytokine staining, gluten-specific T cells did not produce IL-17 or IL-22, but rather IL-21 [44], indicating that these cells share features with follicular helper T cells. In the current study, we found few cells, of which almost all were tetramer-positive cells, that produced relatively large amounts of transcripts for cytokines such as IFN-γ and IL-21. This expression pattern is in accordance with the transcriptional burst of cytokines [45], and in accordance with published bulk RNA-seq study where IL-21 was found to be differentially over-expressed in gluten-specific T cells [25]. To note, in bulk RNA-seq data where the averaged expression of many cells is measured, the expression level of cytokine genes is low. This is consistent with our findings in the single-cell data where transcripts for cytokines were found in few cells, but each cell expressed relatively many transcripts. Compared with tetramer-negative cells, the global transcriptional profiles of tetramer-positive cells showed that the gluten-specific cells had a distinct profile, showing features of either Th1 or Tfh cells. It has been suggested that gluten-specific T cells may interact with plasma cells in the gut of celiac disease patients [25]. IL-21 is a key cytokine for plasma cells that are expanded in the celiac lesion. A recent study suggested that plasma cells may be the main actor of presenting gluten peptides to T cells in the small intestinal lamina propria of active celiac lesion [46].

In a recently published bulk RNA-seq study where gluten-specific T cells were sorted with the same tetramers from duodenal biopsies of five celiac disease patients, 318 genes were found to be differentially expressed between tetramer-positive and tetramer-negative T cells [25]. The DEGs in this bulk dataset was massively dominated by down-regulated genes in tetramer-positive T cells, where 80% were differentially down-regulated. Only 12 genes were found to be differentially expressed in both the bulk and single-cell RNAseq studies. This seemingly small overlap of DEGs is to a certain degree due to the differences in terms of method of testing, statistical power and consequently the threshold applied. Nevertheless, among genes that were differentially regulated in both bulk and single-cell data, we found upregulation of TCR V-genes that are characteristic of gluten-specific T cells (*TRAV26-1*, *TRBV7-2*); overexpression of regulators of T-cell coactivation (CD30L and PD-1 encoded by *TNFSF8* and *PDCD1* respectively); markers of T-cell activation such as the upregulation of MHC class II genes and downregulation of L-selectin (*SELL*); downregulation of the Th17 marker CD26 (*DPP4*); as well as downregulation of cytotoxic genes (*CTSW*, *GZMK*) in tetramer-positive cells. Thus, main conclusions drawn from our single-cell data are supported by bulk data, although the exact gene expression pattern differs.

Single-cell TCR sequencing has shown that gluten-specific T cells are clonally expanded [28]. Likewise, we found a high degree of clonal expansion of tetramer-positive cells in our data. Given the small sampling of total 328 tetramer-positive cells from four different patients where TCR information was successfully retrieved with TraCer, it is noticeable that 47% of tetramer-positive cells shared at least one identical TCR chain with another cell in the same population. Interestingly, we have found that the transcriptional profiles are more similar between cells belonging to the same clonal origin, compared with clonally unrelated tetramer-positive cells from the same individual. Similar findings were made in other settings [24, 47–49].

Despite decades of treatment with gluten-free diet, gluten-specific T cells were found to persist for 20 years in celiac disease patients [11]. Removal or re-programming of gluten-specific cells is thus necessary for a complete cure from the disease. Unbiased multi-parameter studies, including the current single-cell RNA-seq survey of gluten-specific T cells provides the knowledge base for finding unique targets for the removal of gluten-specific T cells as a curative therapeutic option for celiac disease. In our study, we found marked upregulation of several apoptosis-related genes, such as FAS, TRAIL and Caspase 2 in tetramer-positive cells, possibly due to in vivo activation by gluten antigens. These findings encourage the use of activation induced cell death for the removal of gluten-specific T cells, as suggested in [50].

## Supporting information

S1 Table Basic information of patients. (XLSX)

S2 Table Summary on number of cells in each step of analysis. (XLSX)

S3 Table Differentially expressed genes (DEG) with fold change larger than 1.5 (|Log2FC|>0.58) between tetramer-positive and tetramer-negative T cells. (XLSX)

S4 Table Top enriched gene sets in gene set enrichment analysis. (XLSX)

S5 Table List of genes used as signatures for T helper cell subset. (XLSX)

S6 Table Results of TCRs reconstructed by TraCer. (XLSX)

S1 Fig Flow plots of sorting of tetramer-positive effector memory T cells (tet+) and tetramer-negative (tet-) effector memory T cells. The red gatings denote the two populations of cells that were sorted and used in downstream single cell RNA-seq analysis. PBMC stained with PE-conjugated tetramers and antibodies prior to enrichment of PE-stained cells were used for setting the gates.
(PDF)

S2 Fig Criteria for cell selection in quality control. Violin plots show the distribution of cells for each of the four criteria used for quality control. Cells that fulfilled each of the following criteria were included in the downstream analysis: (A) number of reads > 100,000 (B) number of detected genes ranged from 1,800 to 15,000 (C) proportion of reads mapped to mitochondrial genes < 12.5% (D) Mapping rate > 30%.
(PDF)

S3 Fig UMAP plots show batch effect after data integration. (A) UMAP plot of 739 cells from peripheral blood of four untreated celiac disease patients, colored by cell specificity, split by patient. (B) UMAP plot of 326 cells from peripheral blood of patient CD1615, colored by cell specificity, split by plate.
(PDF)

S4 Fig UMAP plot of 964 effector memory CD4^+^ T cells from peripheral blood of two healthy donors and four untreated celiac disease patients. HC: effector memory CD4^+^ T cells from healthy donors; Tet+T: sorted HLA-DQ2:gluten-tetramer-positive effector memory CD4^+^ T cells from CD patients; Tet-T: tetramer-negative effector memory CD4^+^ T cells from CD patients.
(PDF)

S5 Fig Volcano plot of differentially expressed genes between tetramer-positive and tetramer-negative T cells. All genes are plotted with Log2(fold change) along the x-axis and log10(p-value) along the y-axis. Dashed lines are drawn at log2FC = 0.58 or -0.58, and at P = 0.00164 (equivalent to FC = 1.5 and FDR = 0.05). Differentially expressed genes with FDR > 0.05 and FC > 1.5 are colored in red. The identities of some selected differentially expressed genes are shown.
(PDF)

S6 Fig Tetramer-positive T cells display phenotypes of activated effector memory T cells with helper functions. UMAP of 739 cells colored by VISION score for signature genes of T cell differentiation, where cells with higher score have higher expression of genes characterizing naïve CD4^+^ T while those with lower scores have expression of genes characterizing the effector memory CD4^+^ T cell. The gene set used for calculating the signature scores were from [23] in Molecular Signature Database (MsigDB) C7: immunologic signature. Signature scores for cells were summarized by cell specificity in the violin plot. Tet+: tetramer-positive T cells; tet-: tetramer-negative T cells.
(PDF)

## Abbreviations

CD: celiac disease
DEGs: differentially expressed genes
FDR: false discovery rate
HLA: human leukocyte antigen
MHC: major histocompatibility complex
PBMC: peripheral blood mononuclear cells
PCA: principal component analysis
PCs: principal components
SNN: shared nearest neighbor
TCR: T-cell receptor
TCRα: T cell receptor α chain
TCRβ: T cell receptor β chain
TPM: transcripts per million
UMAP: uniform manifold approximation and projection
