# Correction: Differential expression profile of gluten-specific T cells identified by single-cell RNA-seq

**DOI:** 10.1371/journal.pone.0270444

**Published:** 2022-06-21

**Authors:** The PLOS ONE Staff

The second author’s name is spelled incorrectly. The correct name is: Łukasz Wyrożemski. The correct citation is: Yao Y, Wyrożemski Ł, Lundin KEA, Sandve GK, Qiao S-W (2021) Differential expression profile of gluten-specific T cells identified by single-cell RNA-seq. PLoS ONE 16(10): e0258029. https://doi.org/10.1371/journal.pone.0258029. The publisher apologizes for the error.
