# Supplementary figures and images for: Differential expression profile of gluten-specific T cells identified by single-cell RNA-seq
Source: PLoS One. 2021 Oct 7;16(10):e0258029. doi: 10.1371/journal.pone.0258029 (PMC8496852; doi:10.1371/journal.pone.0258029)

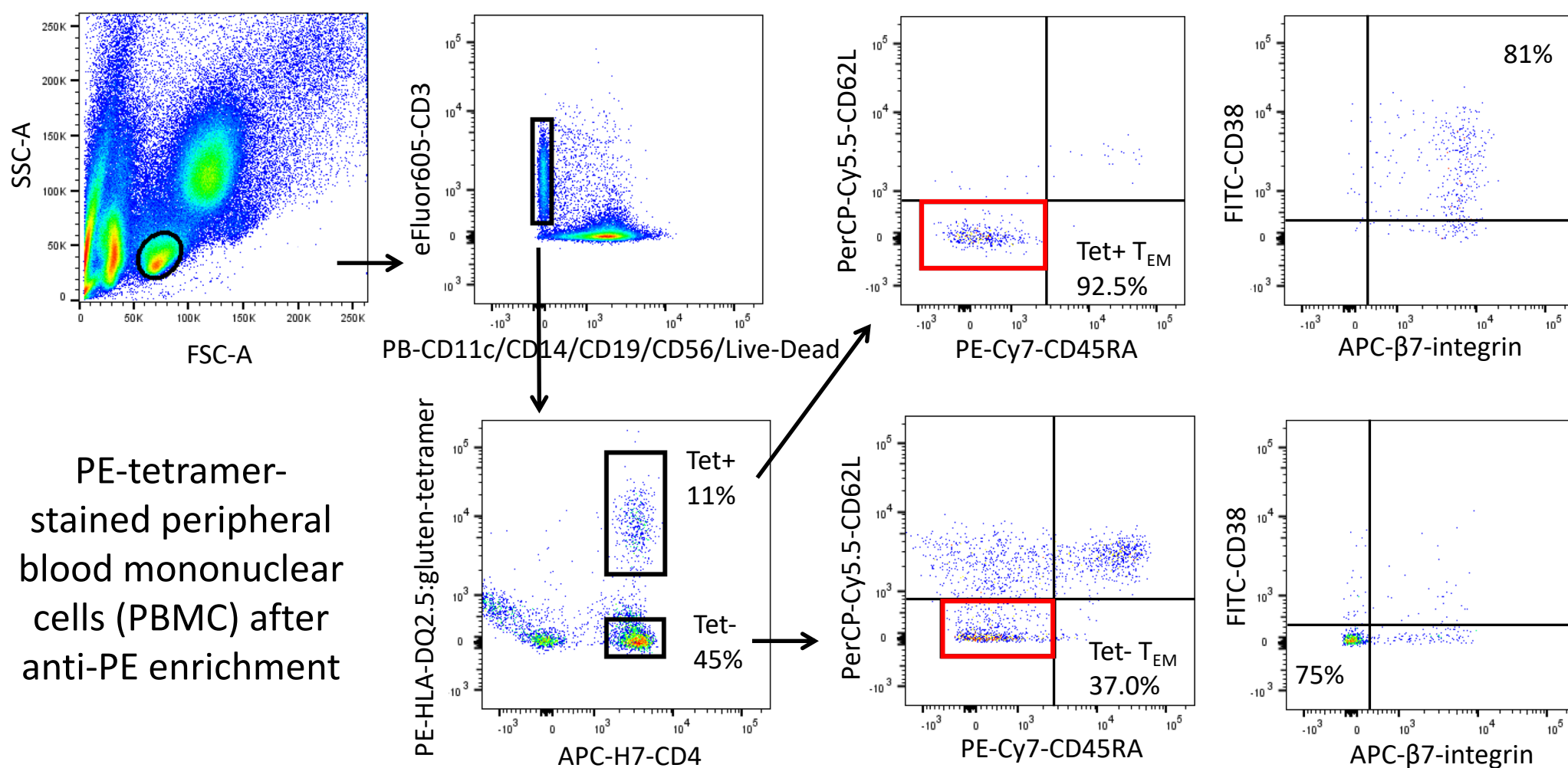

Gating strategies  
by using PE-  
tetramer-stained  
PBMC without  
enrichment

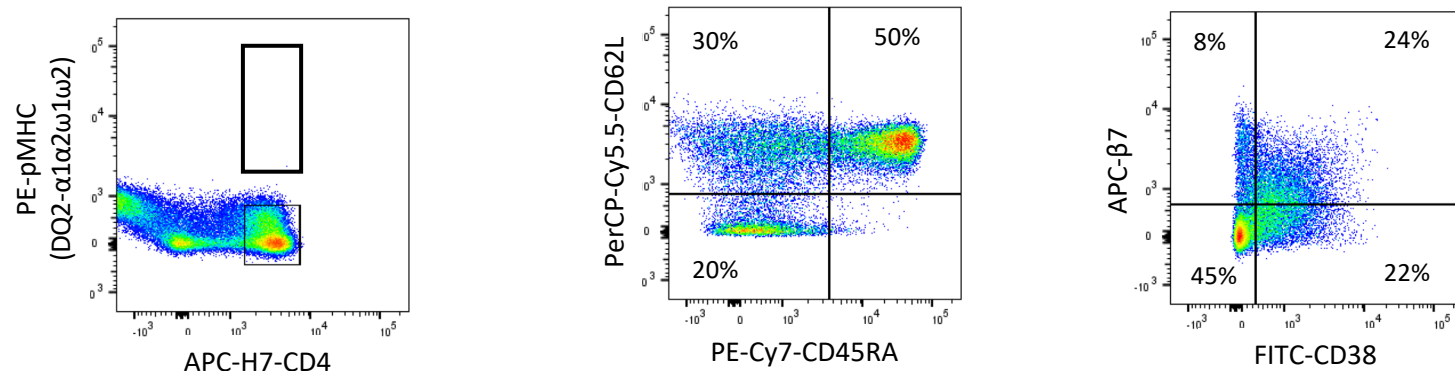

Supplement: S1 Fig — The red gatings denote the two populations of cells that were sorted and used in downstream single cell RNA-seq analysis. PBMC stained with PE-conjugated tetramers and antibodies prior to enrichment of PE-stained cells were used for setting the gates. (PDF) [file pone.0258029.s007.pdf]

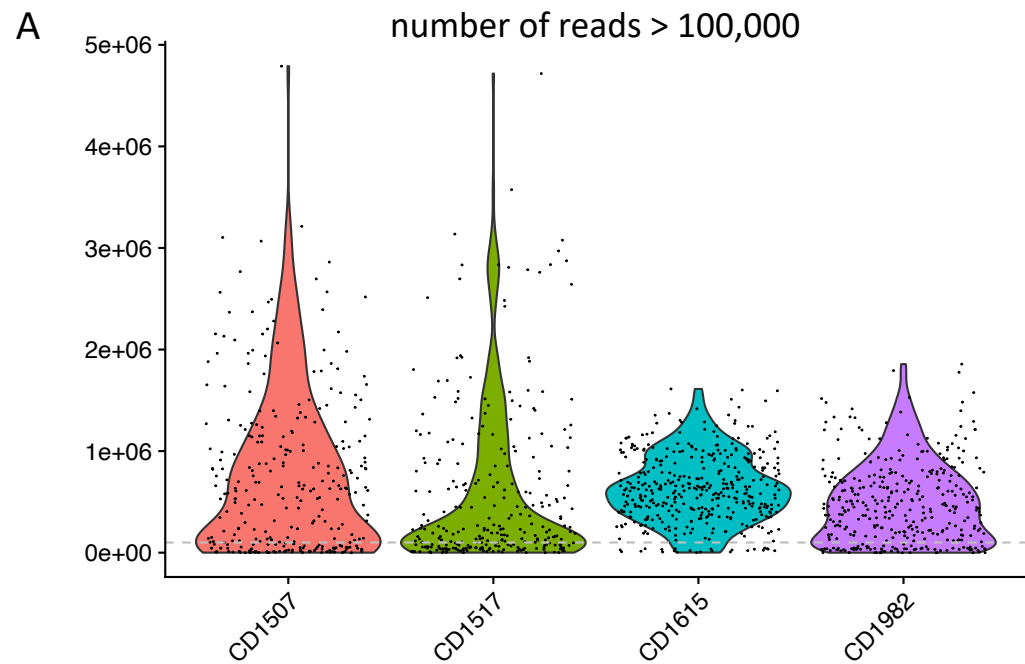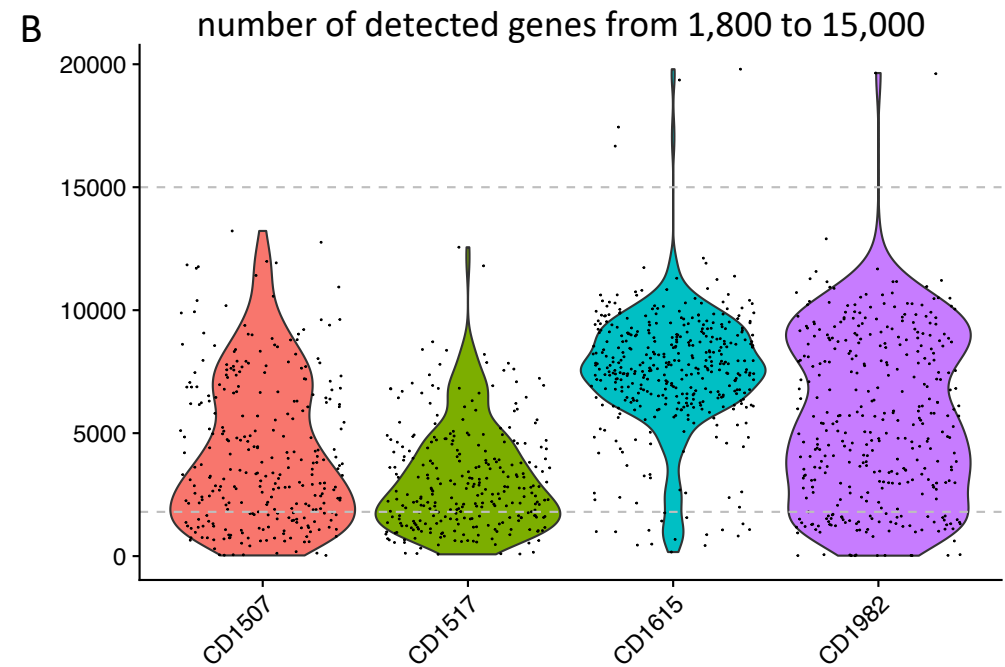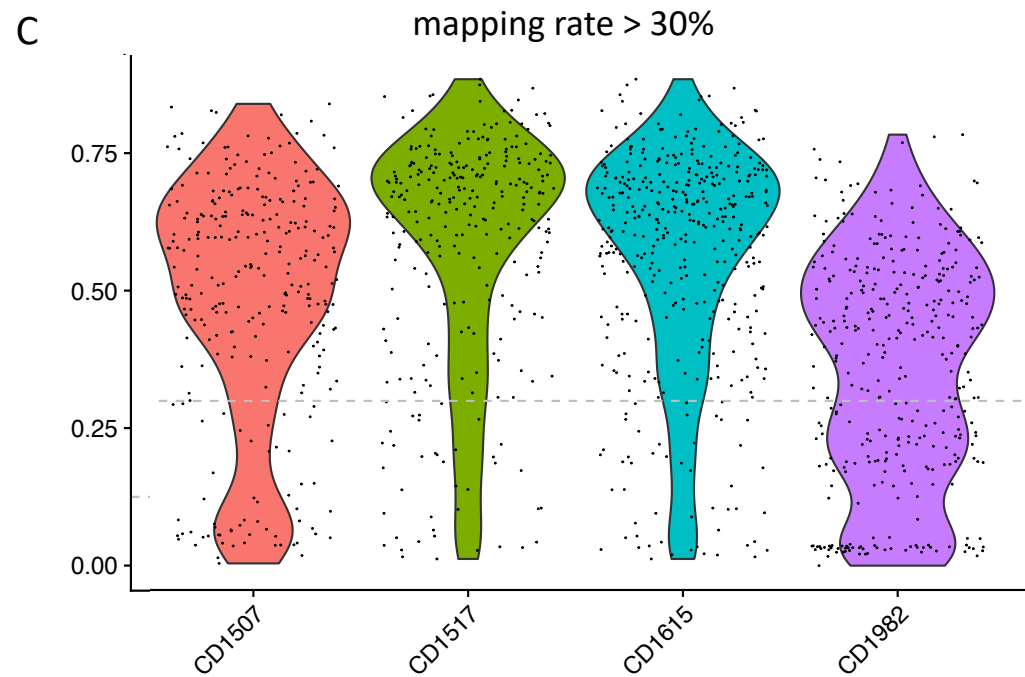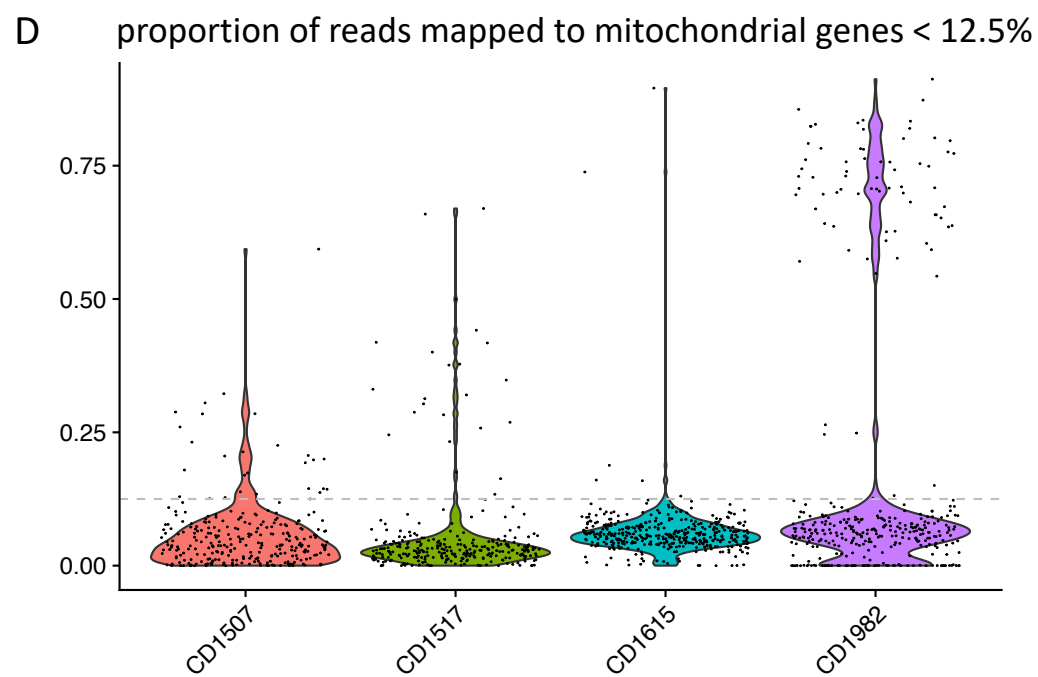

Supplement: S2 Fig — Violin plots show the distribution of cells for each of the four criteria used for quality control. Cells that fulfilled each of the following criteria were included in the downstream analysis: (A) number of reads > 100,000 (B) number of detected genes ranged from 1,800 to 15,000 (C) proportion of reads mapped to mitochondrial genes < 12.5% (D) Mapping rate > 30%. (PDF) [file pone.0258029.s008.pdf]

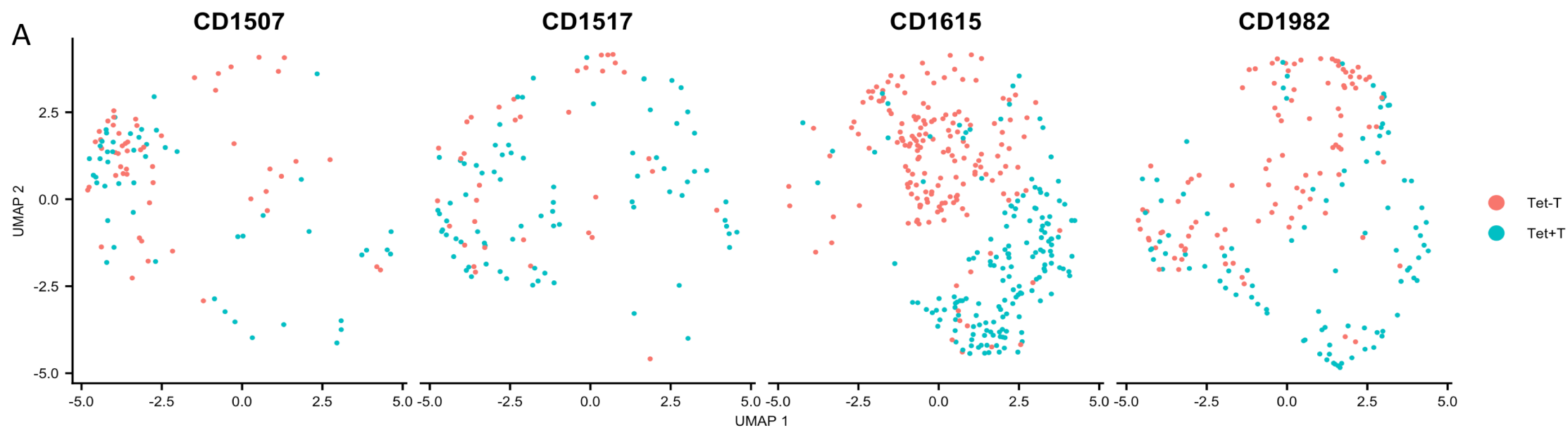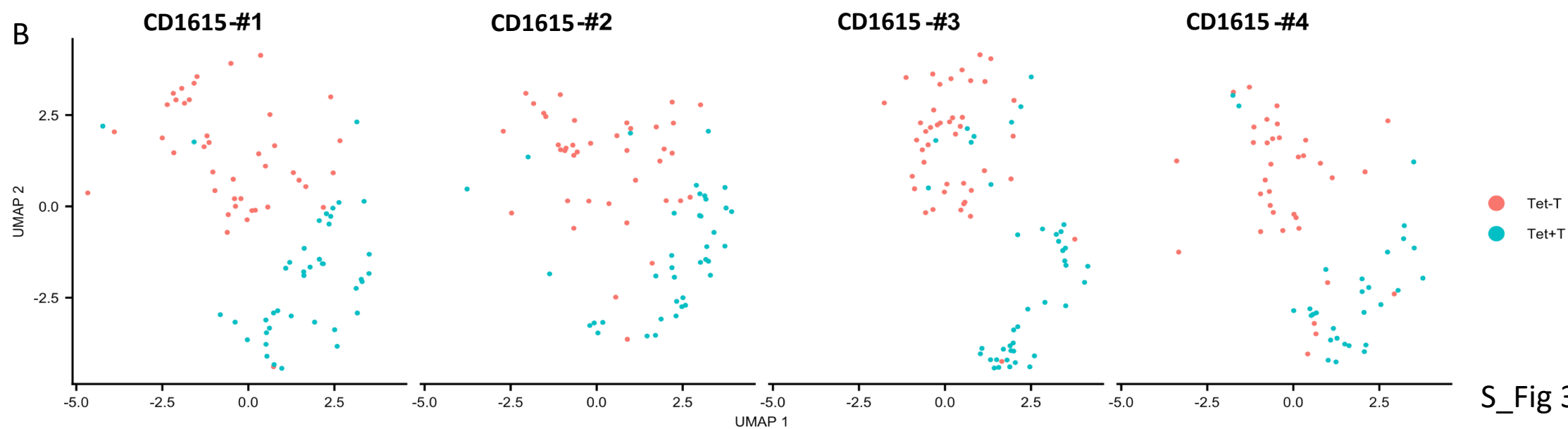

Supplement: S3 Fig — (A) UMAP plot of 739 cells from peripheral blood of four untreated celiac disease patients, colored by cell specificity, split by patient. (B) UMAP plot of 326 cells from peripheral blood of patient CD1615, colored by cell specificity, split by plate. (PDF) [file pone.0258029.s009.pdf]

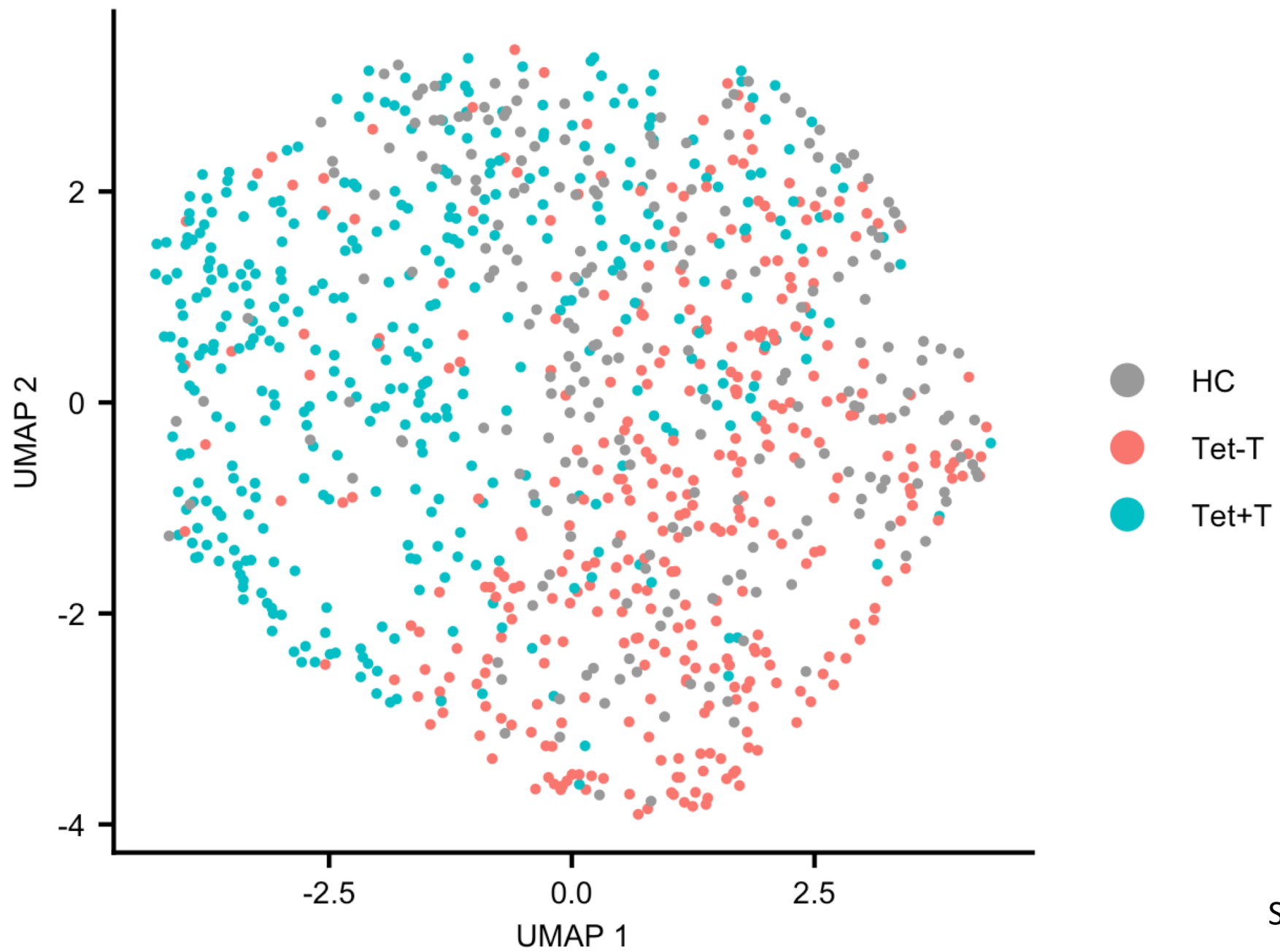

S\_Fig 4

Supplement: S4 Fig — HC: effector memory CD4+ T cells from healthy donors; Tet+T: sorted HLA-DQ2:gluten-tetramer-positive effector memory CD4+ T cells from CD patients; Tet-T: tetramer-negative effector memory CD4+ T cells from CD patients. (PDF) [file pone.0258029.s010.pdf]

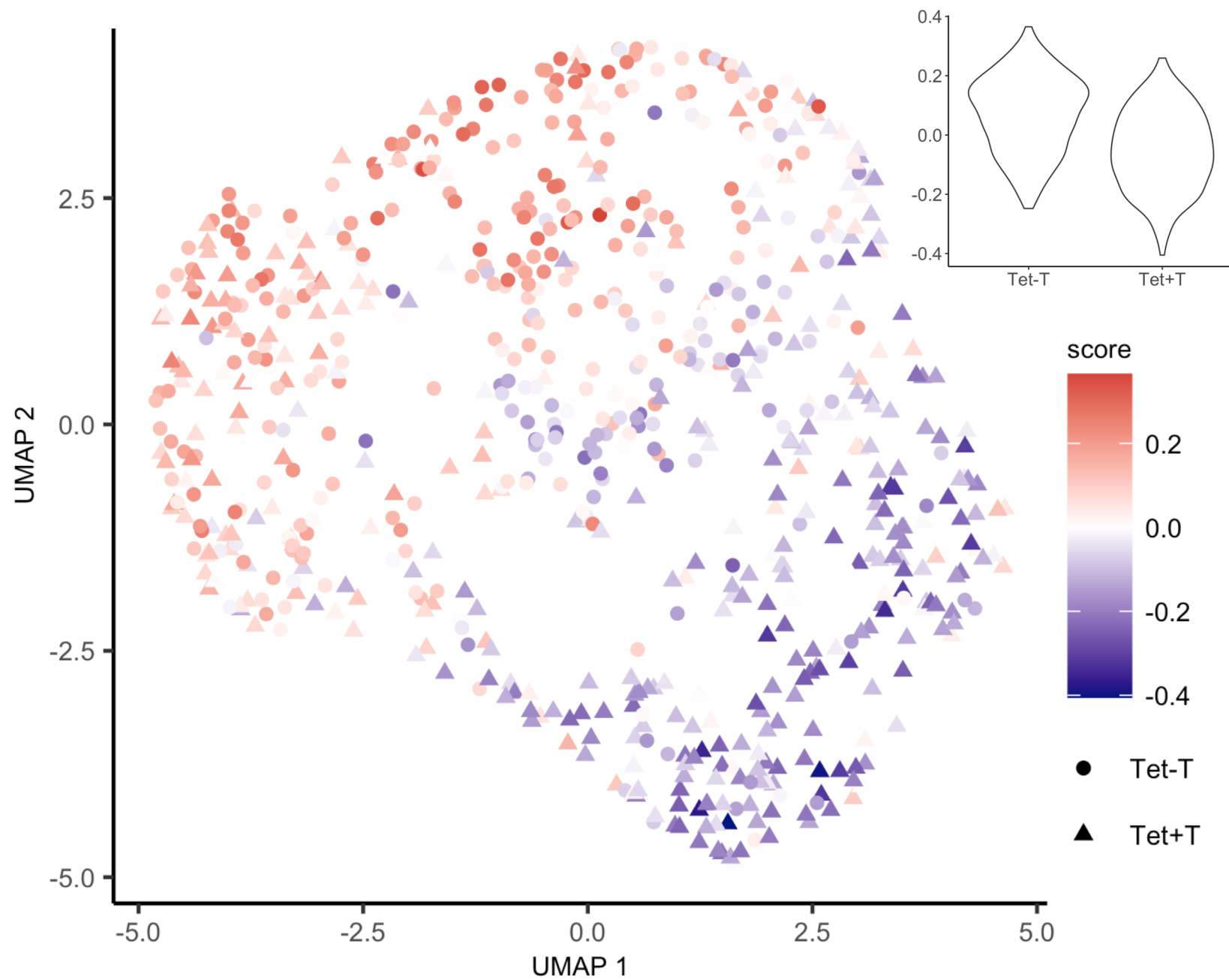

S\_Fig 6

Supplement: S6 Fig — UMAP of 739 cells colored by VISION score for signature genes of T cell differentiation, where cells with higher score have higher expression of genes characterizing naïve CD4+ T while those with lower scores have expression of genes characterizing the effector memory CD4+ T cell. The gene set used for calculating the signature scores were from [23] in Molecular Signature Database (MsigDB) C7: immunologic signature. Signature scores for cells were summarized by cell specificity in the violin plot. Tet+: tetramer-positive T cells; tet-: tetramer-negative T cells. (PDF) [file pone.0258029.s012.pdf]
