# Supplementary material for: Differential expression profile of gluten-specific T cells identified by single-cell RNA-seq
Source: PLoS One. 2021 Oct 7;16(10):e0258029. doi: 10.1371/journal.pone.0258029 (PMC8496852; doi:10.1371/journal.pone.0258029)
Supplement: S5 Fig — All genes are plotted with Log2(fold change) along the x-axis and log10(p-value) along the y-axis. Dashed lines are drawn at log2FC = 0.58 or -0.58, and at P = 0.00164 (equivalent to FC = 1.5 and FDR = 0.05). Differentially expressed genes with FDR > 0.05 and FC > 1.5 are colored in red. The identities of some selected differentially expressed genes are shown. (PDF) [file pone.0258029.s011.pdf]

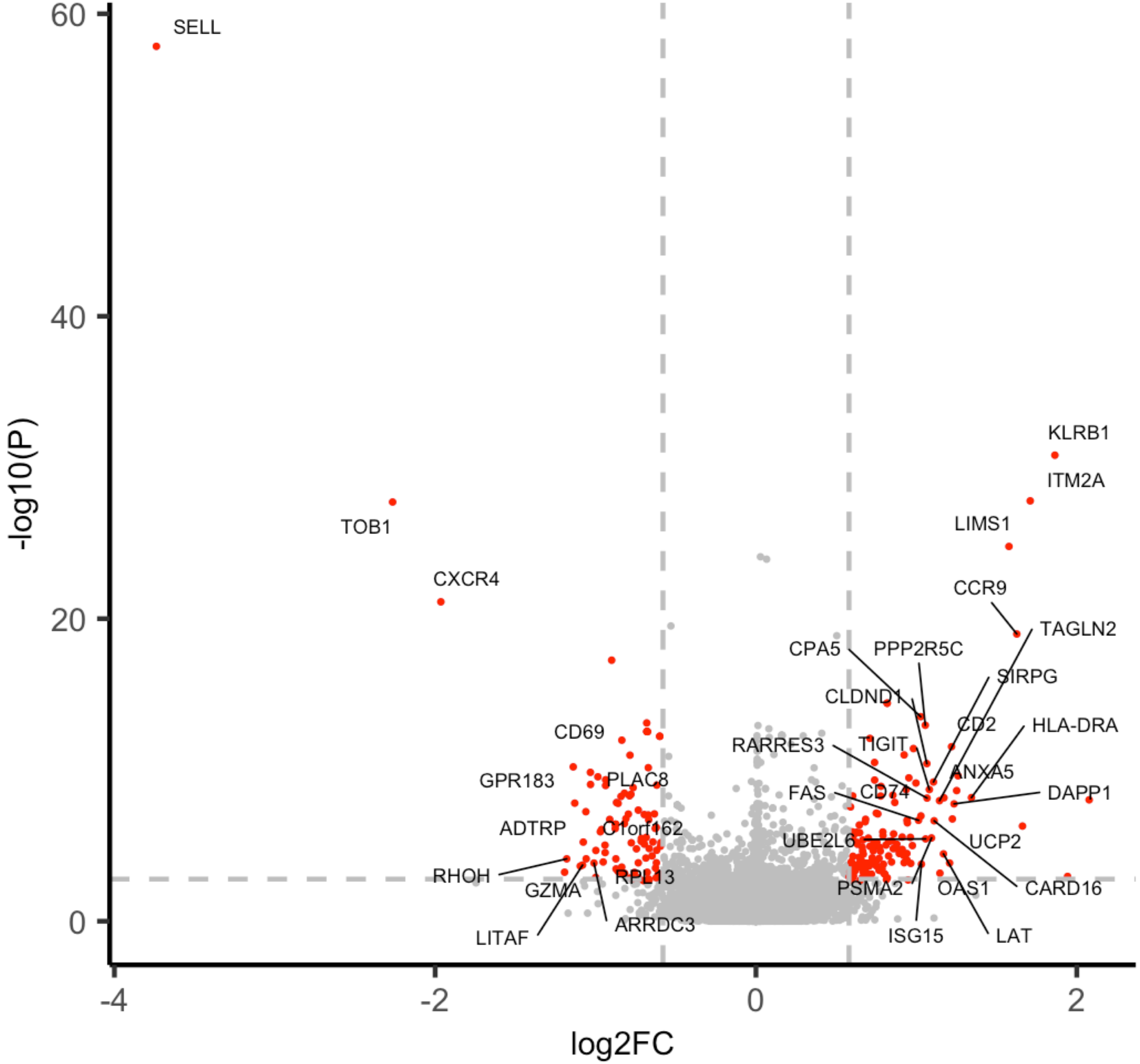
SELL
TOB1
CXCR4
CD69
GPR183
PLAC8
ADTRP
C1orf162
RHOH
GZMA
RPL13
LITAF
ARRDC3
KLRB1
ITM2A
LIMS1
CCR9
TAGLN2
CPA5
PPP2R5C
SIRPG
CLDND1
CD2
HLA-DRA
RARRES3
TIGIT
ANXA5
FAS
CD74
DAPP1
UBE2L6
UCP2
PSMA2
OAS1
CARD16
ISG15
LAT
60
40
20
0
-log10(P)
-4
-2
0
2
log2FC

S_Fig 5
